# Deep reinforcement learning for time-critical wilderness search and rescue using drones

**DOI:** 10.3389/frobt.2024.1527095

**Published:** 2025-02-03

**Authors:** Jan-Hendrik Ewers, David Anderson, Douglas Thomson

**Affiliations:** Autonomous Systems and Connectivity, University of Glasgow, Glasgow, United Kingdom

**Keywords:** reinforcement learning, search planning, mission planning, autonomous systems, wilderness search and rescue, unmanned aerial vehicle, machine learning

## Abstract

Traditional search and rescue methods in wilderness areas can be time-consuming and have limited coverage. Drones offer a faster and more flexible solution, but optimizing their search paths is crucial for effective operations. This paper proposes a novel algorithm using deep reinforcement learning to create efficient search paths for drones in wilderness environments. Our approach leverages *a priori* data about the search area and the missing person in the form of a probability distribution map. This allows the policy to learn optimal flight paths that maximize the probability of finding the missing person quickly. Experimental results show that our method achieves a significant improvement in search times compared to traditional coverage planning and search planning algorithms by over 160% , a difference that can mean life or death in real-world search operations Additionally, unlike previous work, our approach incorporates a continuous action space enabled by cubature, allowing for more nuanced flight patterns.

## 1 Introduction

Wilderness Search and Rescue (WiSAR) operations in Scotland’s vast and often treacherous wilderness pose significant challenges for emergency responders. To combat this, Police Scotland Air Support Unit (PSASU) and Scottish Mountain Rescue (SMR) regularly use helicopters to assist in search operations (Carrell, 2022). However, the deployment of helicopters can be slow, especially in the Scottish Isles where the PSASU’s Eurocopter EC135 based in Glasgow can take multiple hours to arrive. Additionally, operating helicopters is extremely costly.

Drones, also known as unmanned aerial vehicles, offer a cost-effective and agile solution for aerial search. Both PSASU and SMR are placing small fleets of drones around Scotland for rapid deployment in a WiSAR scenario. These fleets will never replace the requirement for a helicopter due to the inherent lifting disparities between the two platforms but will ensure that the search can begin as soon as possible.

The current approach to flying drones for PSASU is the pilot-observer model where two personnel are required at a minimum per drone. In this setup, the observer is in charge of maintaining visual line of sight at all times whilst the pilot can fly the drone and inspect the live camera feed. Koester et al. (2004) identifies that a foot-based searcher has a higher detection rate when not in motion, similar to the behaviour exhibited by pilots (Ewers et al., 2023b). The cognitive load of being in-motion whilst searching is evidently a barrier for pilots and offloading one aspect of this may lead to efficiency gains.

To address this challenge and to also free up precious manpower, search planning algorithms are employed to optimize drone flight paths (Lin and Goodrich, 2009). While Deep Reinforcement Learning (DRL) has not been explored in this context, its success in other domains, such as video games and drone racing (Mnih et al., 2015; Kaufmann et al., 2023), suggests its potential for improving WiSAR search planning. The ability of DRL to generalize the problem and make decisions that maximise future gains based on extensive training allows it to provide unique solutions to the problem.

WiSAR search planning is an information rich task and the effective utilization of prior information - such as the place last seen, age, and fitness levels of the missing person - is critical. This information can be used to generate a Probability Distribution Map (PDM), which describes the probability of detecting the missing person at a given location and informs the search stage of the mission. Without this *a priori* information the search problem converts into either a coverage or exploration problem. The former assumes a uniform PDM whilst the later develops an understanding of the environment in real-time. There are a multitude of algorithms that can generate the PDM (Ewers et al., 2023a; Hashimoto et al., 2022; Šerić et al., 2021) and as such it can be assumed to be a known quantity during search planning.

The core contributions of this research to the field are thus as follows:

• We propose a novel application of DRL to the search planning problem which can outperform benchmark algorithms from the literature (Lin and Goodrich, 2009; Subramanian et al., 2020). This apporach leverages *a priori* information about the search space without limiting its field of view and thus reducing performance.
• We propose the use of a continuous PDM, as opposed to an image-based one (Lin and Goodrich, 2009; Subramanian et al., 2020), to prevent undesired noisy rewards (Guo and Wang, 2023; Fox et al., 2017). This further empowers the policy to use a continuous action space which greatly increases the degrees of freedom over the benchmark algorithms from the literature.
• A framework for calculating the accumulated probability over the search path through cubature (Ewers et al., 2024) is introduced. A different formulation for this calculation from the literature is required due to the use of the continuous PDM and action space.

Related work is discussed in Section 2, and the methodology is presented in Section 3. Results are shown in Section 4, and a conclusion is drawn in Section 5.

## 2 Related work

Coverage planning algorithms have been around for decades (Galceran and Carreras, 2013) in various forms with the most well-known, and intuitive, being the parallel swaths (also known as lawnmower or zig-zag) pattern. This guarantees complete coverage of an entire area given enough time. However, for WiSAR applications, reducing the time to find is substantially more important whilst also dealing with endurance constrained systems like drones.

Lin and Goodrich (2009) approach the search planning problem by using a gradient descent algorithm in the form of Local Hill Climbing (LHC) that can advance into any of the surrounding eight cells. However, LHC alone is not sufficient because Waharte and Trigoni (2010) found that this class of algorithm does not perform well due to their propensity in getting stuck around local maxima. For this reason Lin and Goodrich (2009) introduces the notion of global warming to break out of local maxima. This raises the zero probability floor sequentially a number of times, storing the paths and then reassessing them given the original PDM. Through this, and a convolution-based tie-breaking scheme, LHC_GW_CONV (local hill climb, global warming, convolution) is shown to have very favourable results. However, only the adjacent areas are considered at every time step.

In order to consider the area as a whole, sampling-based optimisation approaches have been applied to the problem. Morin et al. (2023) uses ant colony optimisation with a discrete PDM and Ewers et al. (2023b) uses both genetic algorithm and particle swarm optimisation with a pseudo-continuous PDM. However, due to the nature of sampling-based optimisation problems, they are prone to long computation times to converge on a solution.

A core problem with the previously mentioned algorithms is the inability to consider the PDM as a whole when making decisions. Being able to prioritise long-term goals over short-term gains is a key feature of DRL.

DRL is being used extensively for mission planning such as by Yuksek et al. (2021) who used proximal policy optimisation to create a trajectory for two drones to avoid no-fly-zones whilst tracking towards the mission objective. This approach has defined start and target locations, however the uses of no-fly-zones with constant radius is analogous to an inverted PDM. Peake et al. (2020) uses Recurrent-DDQN for target search in conjunction with A2C for region exploration with a single drone to find missing people. This method does not use any *a priori* information but rather explores the area in real time. This shows that DRL is a suitable approach to the search-over-PDM problem that a WiSAR mission requires.

As highlighted in Section 1, search and exploration planning are very different problems. Exploration planning has seen many different planning algorithms such as in work by Zhang et al. (2024) which uses a partially observable markov decision process and environment discretisation handle exploration and search in near real time. Similarly, Talha et al. (2022) and Peake et al. (2020) use DRL with environment discretisation to explore the environment whilst searching. Ebrahimi et al. (2021) also uses DRL but localizes missing people using radio signal strength indexes without prior knowledge of the searchable domain. These algorithms do not have any *a priori* knowledge available during the planning stage other than what is discovered in real-time; a different problem.

A core aspect of DRL is having a fully observable environment such that the policy can infer why the action resulted in the reward. Thus, being able to represent the PDM effectively is a primary goal. Whilst images can be used as inputs for DRL, as done by Mnih et al. (2015) to play Atari 2600 computer games, the typically large dimension can be prohibitive. However, since PDM generation algorithms most commonly create discrete maps (Šerić et al., 2021; Hashimoto et al., 2022), finding a different representation is required. To go from a discrete to continuous PDM, Lin and Goodrich (2014) uses a gaussian mixture model to represent the PDM as a sum of bivariate Gaussians. This can be easily used to numerically represent the numerous bivariate Gaussian parameters in an array which is a suitable format for a DRL observation.

There are many DRL algorithms to chose from with Proximal Policy Optimisation (PPO) (Schulman et al., 2017) and Soft Actor-Critic (SAC) (Haarnoja et al., 2018) being some of the most prevalent in the literature (Yuksek et al., 2021; Mock and Muknahallipatna, 2023; Xu et al., 2019). Mock and Muknahallipatna (2023) found that PPO performed well for low dimension observation spaces, whilst SAC performed much better for larger ones. The need for a large observation space comes from the fact that the policy would need to have a sense of memory regarding where it had been to encounter *unseen* probability to satisfy the markov decision process that underpins DRL. Mock and Muknahallipatna (2023) found that a recurrent architecture was comparable to including previous states in the observation (also known as frame stacking). This shows that frame-stacking with SAC is a suitable DRL architecture for the current problem.

## 3 Methods

All parameters used in this study can be found in Tables 1, 2.

**TABLE 1:** Simulation parameters used for this study.

Parameter	Value	Units
$N_{\text{gaussian}}$	4	
$\sigma_{i}$	diag(500,500)	
$x_{\text{min}}$ , $y_{\text{max}}$	0	m
$x_{\text{min}}$ , $y_{\text{max}}$	150	m
λ	8	m
$R_{\text{buffer}}$	2.5	m
$N_{\text{waypoint}}$	64	
ϵ	0.1	
$w_{\mathrm{oob}}$	1.0	
$w_{r}$	0.5	
$w_{0}$	0.5	

**TABLE 2:** SAC hyperparameters used for this study from empirical testing. Other variables were kept at the default values from Raffin et al. (2021) v2.1.0.

Hyperparameter	Value
Learning rate	$10^{-6}$
Optimizer	AdamKingma and Ba (2017)
Batch size	1024
Learning starts	8192
Buffer size	$5\times 10^{6}$
Training frequency	10
Gradient steps	50
τ	$10^{-4}$

### 3.1 Modelling

#### 3.1.1 Environment

The low level tasks of control (Tedrake, 2023; Fresk and Nikolakopoulos, 2013; Wang et al., 2023), trajectory generation (Yu et al., 2023), and obstacle avoidance (Richter et al., 2016; Levine et al., 2010; Swinton et al., 2024) can be assumed to be of a high enough standard as to achieve perfect waypoint mission execution performance. The drone within the environment is therefore modelled as a simple heading control model with a constant step size λ . Thus, the position vector $x\in R^{2}$ is updated via.

$$\begin{array}{ll} x_{t+1} & =x_{t}+\lambda \left[\begin{array}{c} \cos u_{t}\\ \sin u_{t} \end{array}\right]\\ & \;u_{t}=\pi \left(a_{t}+1\right) \end{array}$$ (1)

where $a_{t}\in \left[-1,1\right]$ is the policy action at time-step t .

From Equation 1 it is clear that the state $x_{t=T}$ is dependent on the states from t=0 to t=T making this model suitable for formulating the drone’s motion as a markov decision process (MDP). We define the tuple (S,A,P,R) where.

• $s\in S=R^{2}$ is the finite set of states, representing the possible positions of the drone.
• a∈[−1,1] is the action space.
• P:S×A×S→[0,∞] is the unknown transition probability function.
• A:S×A→R is the reward function.

We further define the reward function in Section 3.1.3 and outline the training of the optimal policy $\pi^{*}\left(s|a\right)$ in Section 3.2.

#### 3.1.2 Probability distribution map

In a real WiSAR scenario, algorithms such as Ewers et al. (2023a), Hashimoto et al. (2022), and Šerić et al. (2021) can be employed to generate the PDM given the search mission profile - last place seen, terrain, profile of the lost person, and more. This data is not publicly available in any meaningful quantity and is thus not usable in this scenario. Therefore, as is common within the literature, the PDM is randomly generated for training and evaluation.

**FIGURE 1:**
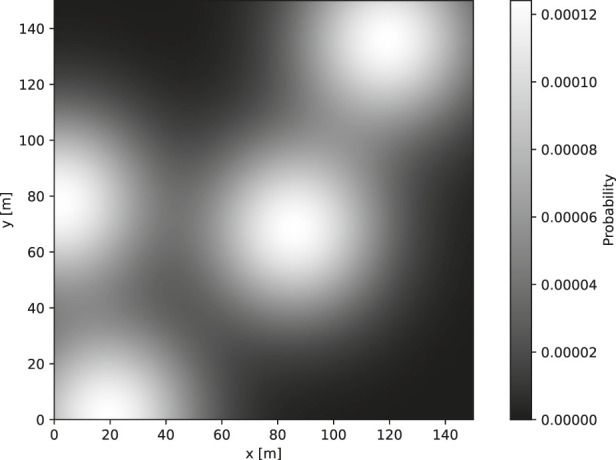
An example multi-modal bivariate Gaussian PDM.

The PDM is modelled as a sum of $N_{\text{gaussian}}$ bivariate Gaussians (Yao et al., 2019) such that a point on the ground at coordinate $x\in R^{2}$ has a probability of containing the missing person.

$$\begin{array}{ll} p\left(x\right) & =\frac{1}{N_{\text{gaussian}}}\overset{N_{\text{gaussian}}}{\underset{i=0}{\sum }}\frac{\exp\left[-\frac{1}{2}{\left(x-\mu_{i}\right)}^{T}\sigma_{i}^{-1}\left(x-\mu_{i}\right)\right]}{\sqrt{4\pi^{2}\det\sigma_{i}}}\\ & \;\forall i\in \left[0,G\right],\mu_{i}∼U\left(\left[x_{\text{min}},x_{\text{max}}\right],\left[y_{\text{min}},y_{\text{max}}\right]\right) \end{array}$$ (2)

where $\mu_{i}$ and $\sigma_{i}$ are the mean location and covariance matrix of the i th bivariate Gaussian respectively. If the bounding area were infinite, that is $x_{\text{min}}=y_{\text{min}}=-\infty$ and $x_{\text{max}}=y_{\text{max}}=\infty$ , then ∑p(x)=1 . However, as can be seen from Figure 1, the area enclosed by the rectangular bounds contain less than this. Section 3.1.3 further discusses how this is handled such that the available probability is normalized.

#### 3.1.3 Reward

As the agent moves a constant distance sm every step, it is assumed that the camera follows this path continuously at a fixed height whilst pointing straight down at all times. Therefore, to represent the *seen area* for a given path at time-step t , the path is buffered by $R_{\text{buffer}}\text{m}$ to give the polygon $h_{t}$ . All probability from the PDM enclosed within $h_{t}$ is then *seen* and denoted by $p_{t}$ . This value, the seen probability, is calculated through

$$I\left(H\right)=\int_{H}p\left(x\right)dH$$

with $H=h_{t}$ . p(x) is from Equation 2. Thusly,

$$p_{t}=I\left(h_{t}\right)$$

**FIGURE 2:**
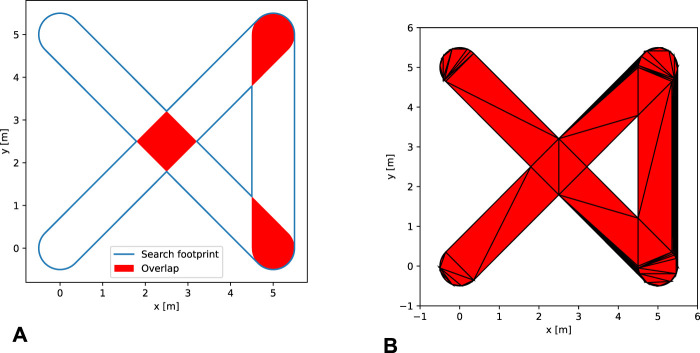
Visualizations of concepts related to the buffered polygon representation of the seen area. **(A)** An example of how the buffered path polygon h automatically deals with re-seen areas. Note that the highlighted areas are just for demonstration and are not a part of the algorithm. **(B)** The polygon from **(A)** triangulated using the Delauney constrained triangulation.

The integral is calculated using a cubature integration scheme (Ewers et al., 2024) with constrained Delaunay triangulation (Chew, 1987) to subdivide H into triangles as seen in Figure 2B.

Other than allowing easy calculation of the accumulated probability, the buffering of the path prevents revisiting of an area contributing the same probability multiple times. This can be seen at the cross-over point (2.5,2.5)m in Figure 2A.

**FIGURE 3:**
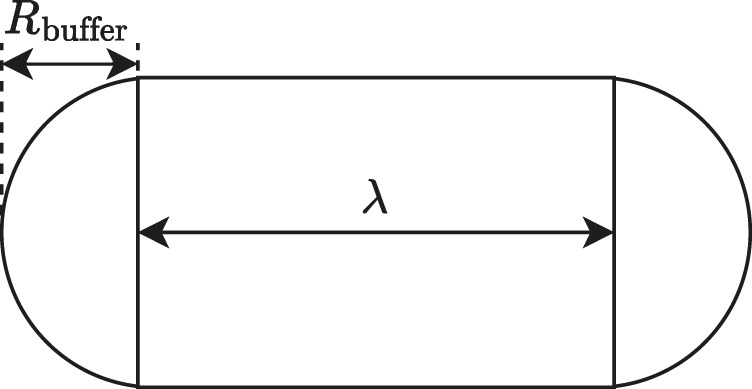
The anatomy of the area calculation of an isolated step of length λ with buffer $R_{\text{buffer}}$ as used in Equation 4.

In order to correlate action to reward, only the additional probability that has been accumulated

$$\Delta p_{t}=p_{t}-p_{t-1}$$ (3)

is used. To normalize this value, the scaling constant k is introduced. This scales $\Delta p_{t}$ by the ratio of the area of an isolated step dm to the area of the total search area $a_{\text{area}}m^{2}$ . This is defined as

$$k=\frac{a_{\text{area}}}{R_{\text{buffer}}\left(\pi R_{\text{buffer}}+2\lambda \right)}$$ (4)

with further spatial definitions from Figure 3.

As highlighted in Section 3.1.2, the enclosed probability by the bounds is not equal to 1. To handle this, $\Delta p_{t}$ is scaled by the total available probability within the search area $p_{A}=I\left(A\right)$ . Combining $p_{A}$ with Equations 3, 4, gives the reward

$$r=\frac{k}{p_{A}}\Delta p_{t}$$

The enclosed probability can then be used to calculate the probability efficiency at time-step t with

$$e_{p,t}=\frac{p_{t}}{p_{A}}$$ (5)

and $e_{p,t}\leq 1$ .

Finally, reward shaping is used to discourage future out-of-bounds actions and to penalize visiting areas of low probability or revisiting previously seen sections. The latter is easily handled by the buffering of the path as seen in Figure 2A, where the areas highlighted in red will contribute no value to the reward resulting in a penalty of $-w_{\mathrm{oob}}$ . The augmented reward $r^{\prime }$ is then defined as

$$r^{\prime }=\left\{\begin{array}{cc} -w_{\mathrm{oob}},\; & x_{t}\notin \left[x_{\text{min}},x_{\text{max}}\right]\times \left[y_{\text{min}},y_{\text{max}}\right]\\ w_{r}r,\; & \Delta p_{t}>\epsilon \\ -w_{0},\; & \text{else} \end{array}\right.$$

### 3.2 Training algorithm

**FIGURE 4:**
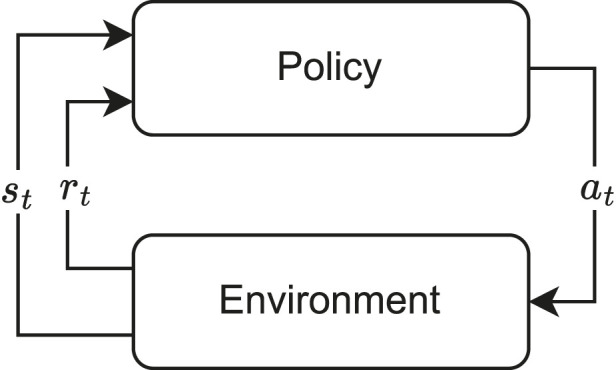
Top-level representation of a typical reinforcement learning data flow. The agent is also commonly referred to as the policy.

SAC is a DLR algorithm particularly effective for continuous control tasks and it addresses the exploration-exploitation dilemma by simultaneously maximizing expected reward and entropy. The interaction of the policy with the environment can be seen in Figure 4. Entropy, a measure of uncertainty, encourages the agent to explore the environment, preventing it from getting stuck in suboptimal solutions.

SAC employs an actor-critic architecture consisting of the following key components:

• Policy Network (Actor): Denoted as $\pi_{\phi }\left(a|s\right)$ , it represents the current policy parameterized by ϕ . SAC is a stochastic algorithm and thus actions are sampled from the policy through $a_{t}∼\pi_{\phi }\left(a_{t}|s_{t}\right)$ .
• Q-function Networks (Critics): SAC uses two soft Q-networks, $Q_{\theta_{1}}\left(s,a\right)$ and $Q_{\theta_{2}}\left(s,a\right)$ , parameterized by $\theta_{1}$ and $\theta_{2}$ respectively. These estimate the expected cumulative reward for taking action a in state s and following the policy thereafter.
• Value Network: While not explicitly maintained, the soft state-value function V(s) is implicitly defined as:

$$V\left(s_{t}\right)=E_{a_{t}∼\pi_{\phi }}\left[Q\left(s_{t},a_{t}\right)-\alpha \log\pi_{\phi }\left(a_{t}|s_{t}\right)\right]$$

The use of two soft Q-networks helps to reduce positive bias in the policy improvement step, a common issue in value-based methods. The soft Q-networks can be trained to minimize the soft Bellman residual:

$$J_{Q}\left(\theta \right)=E_{\left(s_{t},a_{t}\right)∼D}\left[\frac{1}{2}{\left.\left(Q_{\theta }\left(s_{t},a_{t}\right)-\left(r\left(s_{t},a_{t}\right)+\gamma E_{s_{t+1}∼p}\left[V_{\bar{\theta }}\left(s_{t+1}\right)\right]\right)\right)\right)}^{2}\right]$$

SAC incorporates an automatic entropy tuning mechanism (Haarnoja et al., 2019) to adjust the temperature parameter α during training. This allows the algorithm to adapt the degree of exploration based on the policy’s performance. α is learnt by minimizing the following loss before the target network is updated with:

$$J\left(\alpha_{t}\right)=E_{a_{t}∼\pi_{t}}\left[-\alpha_{t}\log\pi_{t}\left(a_{t}|s_{t};\alpha_{t}\right)-\alpha_{t}H\left(\pi_{\theta }\left(\cdot |s_{t}\right)\right)\right]$$

where $H\left(\pi_{\theta }\left(\cdot |s_{t}\right)\right)$ is the entropy of the policy $\pi_{\theta }$ given the state $s_{t}$ .

By automatically tuning α , SAC can maintain an appropriate balance between exploration and exploitation throughout the learning process, adapting to the complexity of the task and the stage of learning.

#### 3.2.1 Policy network

**FIGURE 5:**
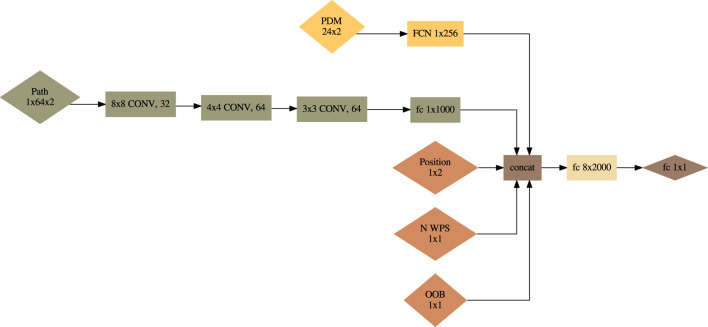
Policy network $\pi_{\phi }$ . $\pi_{\theta ,path}$ uses a 2D CNN architecture from Mnih et al. (2015).

The core of the policy network $\pi_{\theta ,core}$ consists of a fully connected network with $N_{\text{layers}}$ layers, each with a width of $N_{\text{width}}$ . The observation definitions are given in Table 3 resulting in $2N_{\text{waypoints}}+6G+4$ observation inputs. The policy $\pi_{\phi }$ is constructed to handle multiple input observation spaces and is defined in Figure 5. The inner workings of the policy are defined in Algorithm 1.

**TABLE 3:** Definition of the five state observations.

Sub-state	Symbol	Definition
Path	$s_{\text{path}}$	${\left(x\;\|\;0^{2\times N_{\text{waypoints}}-t}\right)}^{T}$
PDM	$s_{\text{PDM}}$	${\left[\mu_{0},\sigma_{0},\ldots ,\mu_{G},\sigma_{G}\right]}^{T}$
Position	$s_{\text{pos}}$	$x_{t}$
Out-of-bounds	$s_{\text{oob}}$	$x_{t}\in \left[x_{\text{min}},x_{\text{max}}\right]\times \left[y_{\text{min}},y_{\text{max}}\right]$
Number of steps	$s_{\text{steps}}$	t

Algorithm 1 Policy Network $\pi_{\phi }$ Input Process.

**Input:** Observations defined in Table 3
**Output:** Action a

Extract PDM latent space $z_{\text{PDM}}\leftarrow \pi_{\theta ,PDM}\left(s_{\text{PDM}}\right)$ ;
Extract path history latent space $z_{\text{path}}\leftarrow \pi_{\theta ,path}\left(s_{\text{path}}\right)$ ;
Concatenate remaining observations and latent spaces $z\leftarrow z_{\text{PDM}}\cup z_{\text{path}}\cup \left[s_{\text{oob}},s_{\text{pos}},s_{\text{steps}}\right]$ ; Sample from policy to get action $a∼\pi_{\theta ,core}\left(z\right)$

## 4 Results

### 4.1 Experimental setup

In order to effectively benchmark the proposed algorithms, two additional baselines are implemented; lawnmower (Galceran and Carreras, 2013) and LHC_GW_CONV (Lin and Goodrich, 2009). These were chosen due to the former being ubiquitous for coverage planning, and the latter being a optimisation-based implementation that struggles to fully explore the PDM. To ensure compatibility in the comparison to the proposed algorithm, the parallel lines for lawnmower are offset by the step size λ and the grid dimensions for LHC_GW_CONV are $\left(x_{\text{max}}-x_{\text{min}},y_{\text{max}}-y_{\text{min}}\right)/\lambda$ . The maximum number of waypoints $N_{\text{waypoint}}$ are converted to a maximum distance $D_{\text{max}}=\lambda N_{\text{waypoint}}$ and the generated paths are truncated at this point.

The results for the algorithm implemented in this research, titled *SAC-FS-CNN* from here on in, is the cumulation of three separate training runs with random starting seeds. This aligns with the best practices outlined by Agarwal et al. (2022) to ensure robust analysis for DRL results. Each model was trained for a minimum of 21 days ( $5\times 10^{8}$ global steps) with 32 workers on a local Ubuntu 22.04 machine with a AMD Ryzen 9 5950X CPU, a NVIDIA RTX A6000 GPU, and 64GB of RAM.

One evaluation of an algorithm involves generating the random PDM, then creating the resultant search path. This is labelled one run. Each algorithm was evaluated at least $5\times 10^{3}$ times and this generated data is base of the following analysis.

### 4.2 Probability over distance (POD)

Maximising the probability efficiency (Equation 5) at all times is critical. This directly correlates to increasing the chances of finding a missing person in a shorter time. It is important for the POD of SAC-FS-CNN to out-perform the benchmark algorithms at all times. If this is not the case, the search algorithm selection becomes dependent on the endurance and mission. However, if the POD is better at all times then one algorithm will be superior no matter the application. To calculate the POD, the probability efficiency is evaluated at

$$d=\frac{\left(N_{\text{steps}}-i\right)D}{N_{\text{steps}}}\forall i\in \left\{N_{\text{steps}},N_{\text{steps}}-1,\ldots ,1,0\right\}$$ (6)

with $N_{\text{steps}}=50$ .

**FIGURE 6:**
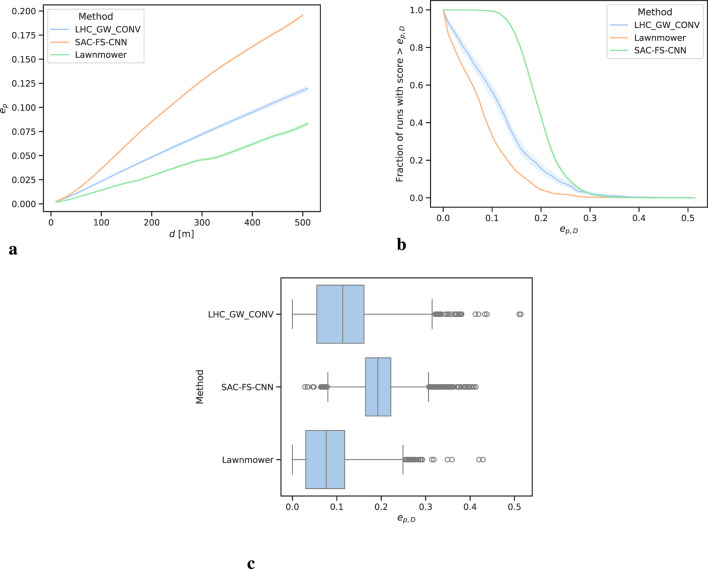
Probability efficiency analysis over all runs. The shaded regions in **(A, B)** show the 95% confidence interval. **(A)** Mean $e_{p}$ with d showing SAC-FS-CNN outperforming the benchmark algorithms at all distances. **(B)** Performance profile of $e_{p,D}$ . SAC-FS-CNN has a close to 100% of runs with $e_{p,D}>0.1$ compared to the < 50% for the other algorithms. **(C)** Median $e_{p,D}$ for all runs. SAC-FS-CNN has a higher median than the other algorithms. LHC_GW_CONV registers large outliers.

From Figure 6A it is clear that SAC-FS-CNN sufficiently outperforms the benchmark algorithms at all distances. This is further highlighted by the $e_{p,D}$ for SAC-FS-CNN at 238% of that of lawnmower, and 158% for LHC_GW_CONV from Table 4. This is corroborated by the median $e_{p,D}$ values in Figure 6C. Notably, however, LHC_GW_CONV has a substantial amount of high $e_{p,D}$ outliers.

**TABLE 4:** Mean POD with the standard deviation as error.

Method	$p_{D}$	$e_{p,D}$	N
LHC_GW_CONV	0.09±0.06	0.12±0.08	$9.3\times 10^{3}$
Lawnmower	0.06±0.04	0.08±0.06	$10\times 10^{3}$
SAC-FS-CNN	0.15±0.04	0.19±0.04	$9.8\times 10^{3}$

Likewise, the performance profile from Figure 6B follows the trend. It can be seen that SAC-FS-CNN has close to 100% of runs with $e_{p,D}>0.1$ and 50% at approximately $e_{p,D}>0.2$ . This aligns with results from Figure 6C and Table 4.

It is of note that LHC_GW_CONV has the largest range of values going from 0.0 to 0.5 whilst SAC-FS-CNN only goes from 0.02 to 0.41. This shows that LHC_GW_CONV can perform very well given the right PDM or poorly given the wrong one. The DLR approach of SAC-FS-CNN, on the other hand, does not suffer from this due to its ability to find a general solution to the problem.

### 4.3 Distance to find (DTF) and percentage found (PF)

Whilst POD shows the theoretical effectiveness of an algorithm, the intended use-case is finding a missing person whilst searching within a bounded area. The mission statement is reducing the time it takes to find the potentially vulnerable person to save lives.

To quantify this requirement, we introduce DTF and PF. The former gives a clear answer on the capabilities on the various algorithms, whilst the latter should align with the POD results from Table 4 for validation.

Firstly, Gumbel-Softmax (Li et al., 2021) is used to sample $N_{\text{samples}}$ positions from the PDM to give the set $\chi \in R^{2\times N_{\text{samples}}}$ containing all samples. The path is then traversed in incremental steps using Equation 6 with $N_{\text{step}}=10^{4}$ . At each step, a euclidean distance check is done from the current position x to each entry in χ with any points within $R_{buffer}$ being marked as seen. The updated set of positions to search for in the next step is then

$$\chi^{\prime }=\left\{\chi_{i}\in \chi :\|x-\chi_{i}\|>R_{buffer}\right\}$$

**FIGURE 7:**
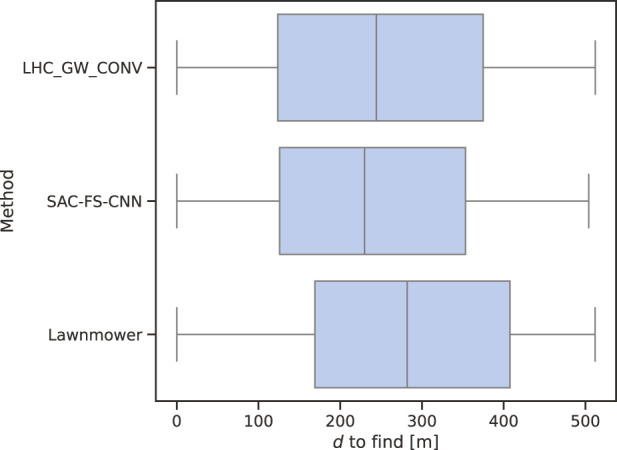
Median d to find. Similarly, the whisker markers across the algorithms shows the broad distribution of results from this experiment. SAC-FS-CNN ultimately has the lowest median score.

From Figure 7, it is clear to see that SAC-FS-CNN outperforms the benchmark algorithms with a lower median DTF as well as a lower inter-quartile range. Table 5 shows that the mean DTF is 15.22% lower than lawnmower, and 4.07% lower than LHC_GW_CONV. This is in line with expectations from the results in Section 4.2. Likewise, the PF values closely match to the $e_{p,D}$ values from Table 4 showing that this test correlates to the theory.

**TABLE 5:** DTF with the standard deviation as error.

Method	PF [%]	Mean DTF [m]	N
LHC_GW_CONV	11.86±0.32	249.37±145.70	$5\times 10^{6}$
Lawnmower	7.77±0.27	282.63±146.62	$5\times 10^{6}$
SAC-FS-CNN	19.00±0.39	239.61±138.16	$10\times 10^{6}$

Whilst SAC-FS-CNN outperforms the benchmarks in the median DTF, it is of note that the variances of the three algorithms are almost identical as shown by the whiskers in Figure 7. This is due to the manner in which the positions are sampled from the random PDM making it likely for there to be a very small subset of positions near the start and end of the path. It is evident that this is the cause because the variances of the three algorithms range from approximately 0 to 512 which is the full simulation distance.

### 4.4 Area efficiency

A path with corners has overlapping regions when considering the buffered path which is evident from Figure 2A. The most efficient path in this formulation is thus a straight line such that the area efficiency is $e_{a,D}=1$ . Using Equation 4, this value is calculated with

$$e_{a,D}=\frac{a_{\text{buffer,D}}}{R_{buffer}\left(\pi R_{buffer}+2D\lambda \right)}$$

where $a_{\text{buffer,D}}$ is the total area of the buffered path, and D is the number of waypoints in the path.

**FIGURE 8:**
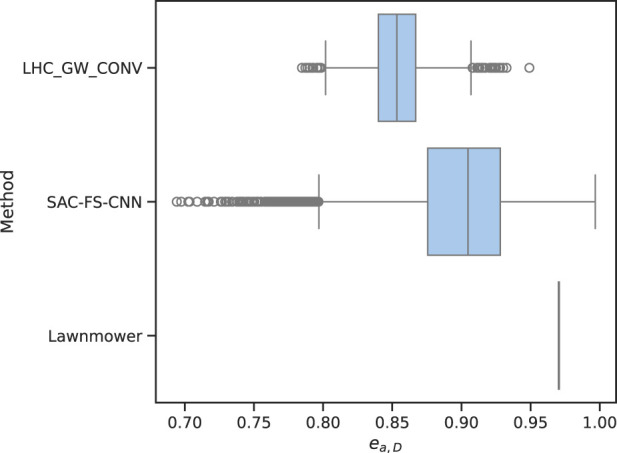
Area efficiency of the three algorithms. Lawnmower has many straight segments which result in a high score of 0.97 and no variance because it does not change. SAC-FS-CNN on the other hand outperforms the other environment-dependent algorithm LHC_GW_CONV in this metric.

The aggregated metrics can be seen in Figure 8 which shows a significant difference in area efficiency between the three methods. The lawnmower method consistently achieves the highest area efficiency values, with a median value of 0.97. This suggests that lawnmower generates paths with minimal overlapping regions within the buffer, resulting in efficient utilization of the search buffer. However, lawnmower has no variance in area efficiency as it is a coverage planning algorithm and as such always generates the same path. In contrast, the SAC-FS-CNN method demonstrates lower area efficiency, with a median value around 0.90. This indicates that SAC-FS-CNN paths tend to have more overlapping areas within the buffer, leading to suboptimal utilization. LHC_GW_CONV method exhibits the lowest area efficiency, with a median value of 0.86 due its inability to make trade-offs now for future gains due to its local hill climbing formulation. This, however, is not the case for LHC_GW_CONV as an infinite-horizon discounted reward is at the core of the SAC algorithm meaning that the current action is taken in order to maximise the future rewards.

## 5 Conclusion

Our research proposed SAC-FS-CNN for search planning in WiSAR operations, leveraging *a priori* information. This was identified as a solution to the challenge of maximizing accumulated probability over a given area due to the powerful capabilities of machine learning to identify patterns and make generalizations in complex tasks.

The results indicate that SAC-FS-CNN can outperform benchmark algorithms in the probability efficiency by up to 250% for lawnmower, and 166% for LHC_GW_CONV. A similar trend is identified when comparing mean DTF with DRL outperforming the aforementioned algorithms by 15.22% and 4.07% respectively. The critical result, however, was that SAC-FS-CNN found 160% more simulated missing people than LHC_GW_CONV. This translates to a substantial advantage in locating missing individuals, potentially saving countless lives during WiSAR operations.

While SAC-FS-CNN exhibits lower area efficiency compared to lawnmower, this trade-off is justified by its superior performance in terms of POD and DTF. Future work could focus on improving area efficiency while maintaining its strong performance in these critical metrics.

The integration of DRL into WiSAR mission planning holds great potential for the future of search, offering a powerful tool with potential to significantly increase the success rate of WiSAR efforts.

## Data Availability

The raw data supporting the conclusions of this article will be made available by the authors, without undue reservation.
