# Supplementary material for: Deep reinforcement learning for time-critical wilderness search and rescue using drones
Source: Front Robot AI. 2025 Feb 3;11:1527095. doi: 10.3389/frobt.2024.1527095 (PMC11831046; doi:10.3389/frobt.2024.1527095)
Supplement: Supplementary file 1 [file DataSheet1.pdf]

Supplementary Material

POLICY NETWORK DESIGN

Search planning is a abstract task compared to point-to-point or coverage planning. Therefore, a parameter sweep for the network architecture was performed to ensure sufficient capabilities. A core aspect of this was the large path history observation with $2N_{\text{waypoint}}$ elements since the policy must be able to learn how to avoid crossing over itself to avoid penalties.

In this sweep four variables were tuned; number of layers N_{layers}, layer width N_{width}, path output feature dimension, and path feature extractor. N_{layers} and N_{width} correlate to the core policy network, and the other feature extractors are left unchanged and can be seen in Fig. 5. The ranges of these sweeps can be seen from Fig. 2. The SAC hyperparameters from the main paper were used for all runs.

There were three path feature extractors that were tested. A standard FCN-based approach (Fig. 1a), a 2D CNN from Mnih et al. (2015) (Fig. 1c), and a 1D CNN variant (Fig. 1b).

In total, 39 runs were completed over 12 days in the same computational environment as outlined in Fig. 4.1. A random sampling strategy was used to ensure broad coverage of the hyperparameter space. Results from this, with a fitted ordinary least squares (OLS) linear regression where applicable, can be seen in Fig. 2.

From the results in Fig. 2a and Fig. 2b it is evident that a deeper, wider network leads to better results. However, Fig. 2b does taper off in performance after $N_{\text{layers}} = 8$ with similar results. The overall number of parameters per result were calculated and plotted in Fig. S3. This confirms the result that a larger policy network is better. A network size of 8×2000 was selected as seen in Fig. 5.

Results from the path output feature dimension in Fig. 2d would be expected to show similar results. However, the OLS has a downward trend implying lower values for being better. Yet the fitted model did not exhibit statistically significant explanatory power, with a p-value of 0.393. For comparison, the p-values for N_{layers} and N_{width} were 0.00522 and 0.00339 respectively and are well below the standard test p-value of 0.05. Thus, a path output feature dimension of 1000 was selected.

Finally, the result for the path feature extraction method in Fig. 2c highlight that the 2D CNN feature extractor yields the highest median $e_{p,D}$. However, the 1D CNN median result is very similar. A p-value was calculated against each distribution to determine if the results were significantly different.

Fig. S4 shows that distribution of 2D CNN results is statistically significantly different from the rest with p-values below 0.05. Therefore, 2D CNN was used as the path feature extractor as seen in Fig. 5.

A limitation of this analysis approach is the need to assume that the results are independent. This is of course not necessarily true as changing the feature extractor might change the requirements for N_{width}. Furthermore, the high p-value score for the path feature extraction method implies that more runs could have been undertaken. It may also imply that this value is not important to the network. However, a more complex analysis is outwith the scope of this study.

REFERENCES

Mnih, V., Kavukcuoglu, K., Silver, D., Rusu, A. A., Veness, J., Bellemare, M. G., et al. (2015). Human-level control through deep reinforcement learning. *Nature* 518, 529–533. doi:10.1038/nature14236

FIGURE CAPTIONS

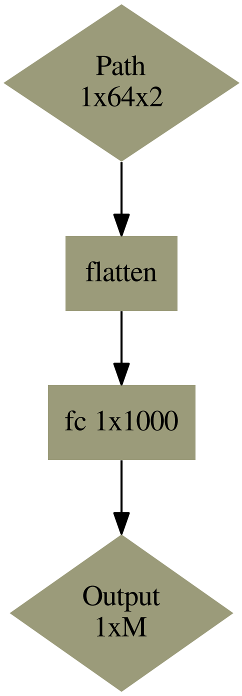

Figure S1a. Single-layer FCN path feature extractor

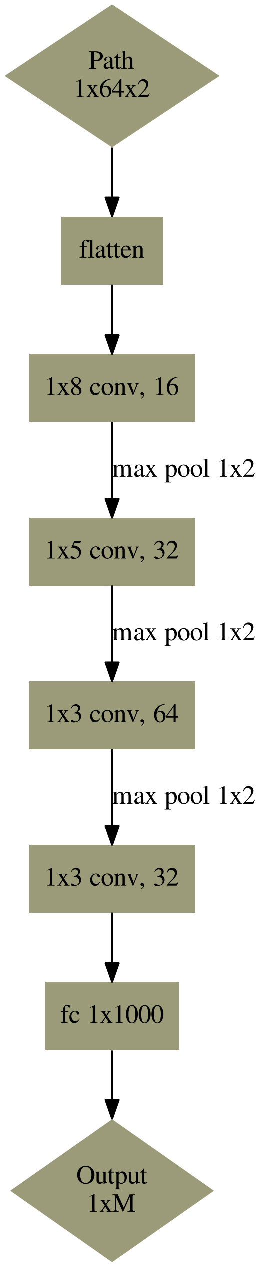

Figure S1b. 1D convolution path feature extractor

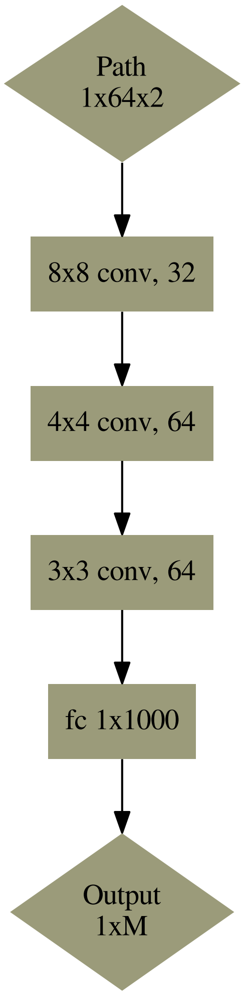

Figure S1c. 2D convolution path feature extractor

Figure S1. Architectures of the three candidate path feature extractors. M is the path output feature dimension.

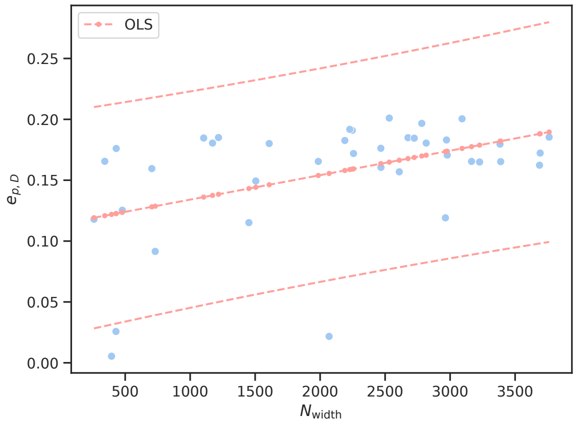

Figure S2a. N_{width}

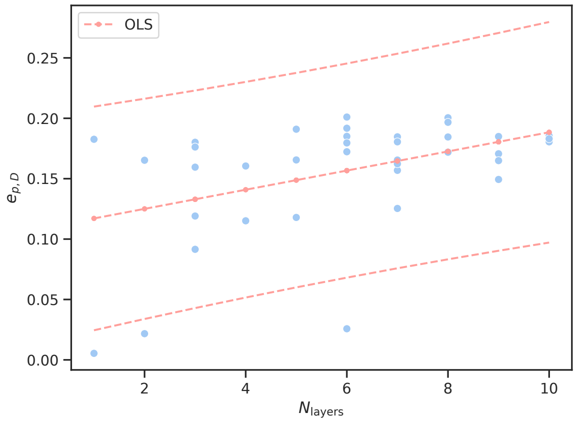

Figure S2b. N_{layer}

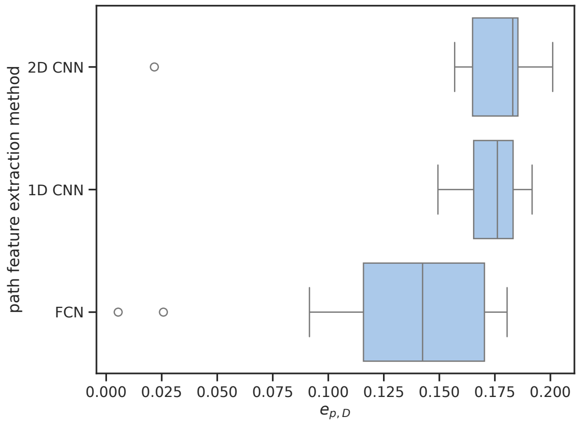

Figure S2c. Path feature extraction method

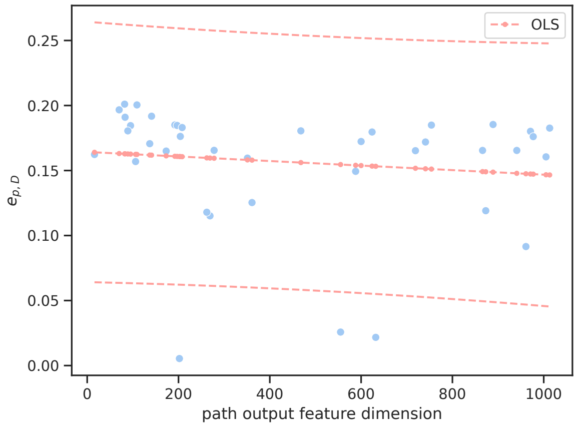

Figure S2d. Path feature extraction method outptu dimension

Figure S2. Ordinary least squares (OLS) linear regression fitted to the $e_{p,D}$ results from the hyperparameter sweep.

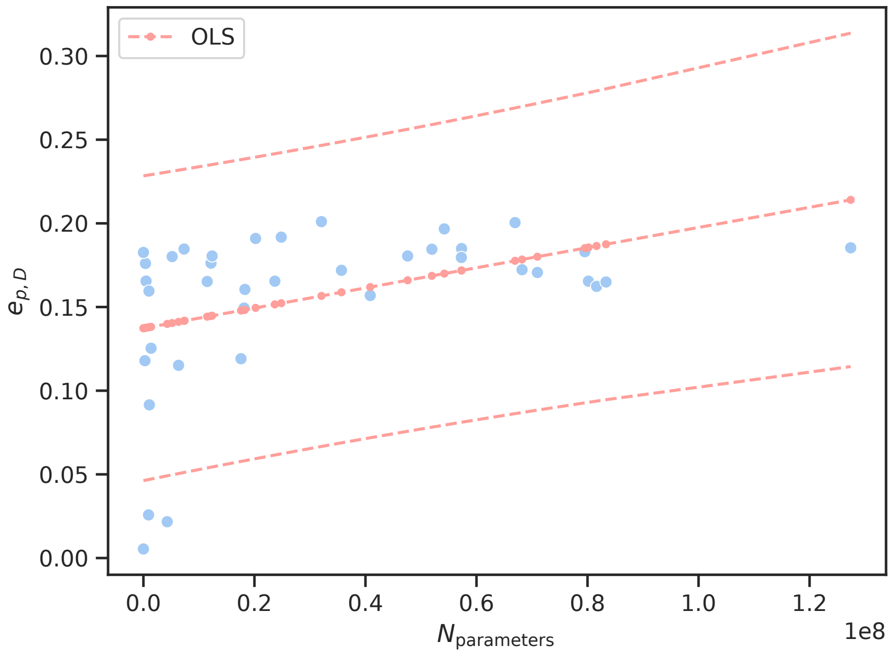

Figure S3. $e_{p,D}$ with the number of parameters within the policy network. This value was derived from Fig. 2a and Fig. 2b.

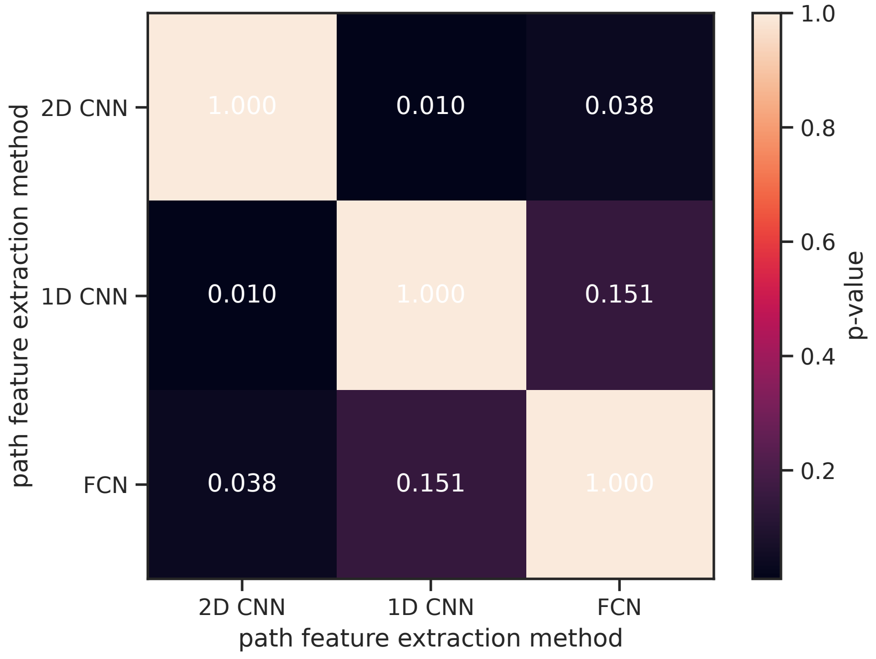

Figure S4. p-value matrix from comparing the result distributions categorized by feature extraction method.
